# Study on the Thermal Condensation Mechanism of Dehydrogenated Polymer (DHP) and Glucuronic Acid

**DOI:** 10.3390/ijms251910533

**Published:** 2024-09-30

**Authors:** Peng Wang, Xu Zhang, Wenyao Peng, Junjun Chen, Junjian An, Guangyan Zhang, Junxian Xie

**Affiliations:** 1 Hubei Provincial Key Laboratory of Green Materials for Light Industry, Hubei University of Technology, Wuhan 430068, China; 2 School of Materials and Chemical Engineering, Hubei University of Technology, Wuhan 430068, China

**Keywords:** dehydrogenated polymer, glucuronic acid, thermal condensation mechanism, biobased wood adhesives

## Abstract

The preparation of traditional wood-based panels mostly uses adhesives such as urea-formaldehyde resin and phenolic resin, which not only consumes petrochemical resources but also releases formaldehyde, posing potential health risks to the human body. Lignin, a natural adhesive in plant cells, is characterized by high reactivity, and it is expected to aid in the development of a new generation of green formaldehyde-free adhesives. However, current studies of lignin adhesives have revealed that while strides have been made in reducing formaldehyde emissions, its residual presence remains a concern, an issue which is compounded by inadequate water resistance. Dehydrogenated Polymer (DHP) has a lignin-like structure and good water resistance, offering a new option for the development of formaldehyde-free adhesives. In this paper, DHP and glucuronic acid were reacted with each other in a simulated hot-pressing environment to obtain DHP-glucuronic acid complex, and then the structure of the complex was characterized by infrared nuclear magnetic resonance to verify whether DHP can be efficiently connected with hemicellulose components under hot-pressing conditions. The results showed that the thermal condensation reaction of DHP and glucuronic acid can generate ester bonds at the Cα position in a simulated hot-pressing environment. This paper explores the thermal condensation mechanism of DHP and glucuronic acid, which is helpful for understanding the bonding process between adhesives and components of wood-based panels in the hot-pressing process, and provides key theoretical support for the design of more sustainable lignin adhesives.

## 1. Introduction

Wood-based panels are widely used in furniture, decoration, packaging, and other industries due to their ease of processing, strong functionality, and high-cost performance [1]. Traditional wood-based panels comprise plates or molded products derived from wood or grass plants which are subjected to mechanical processing to yield various constituent materials, and subsequently bonded and hot-pressed using adhesives. At present, the global annual consumption of wood-based panel adhesives exceeds 30 million tons [2]. Among them, “trialdehyde” adhesives (phenolic resin, urea-formaldehyde resin, and melamine-formaldehyde resin), prepared with formaldehyde as a primary precursor, command prominence within this field. However, harmful substances such as formaldehyde and phenol are released during the production and use of “trialdehyde” adhesives, which cause harm to the environment and human health. The release of low concentrations of formaldehyde (0.6–1.9 ppm) can irritate the nasal cavity and eyes, which causes neurological effects and increases the risk of asthma and allergies. At high concentrations (1.9–10.9 ppm), changes in lung function occur and irritation occurs in the eyes, throat, and skin. Therefore, the development of formaldehyde-free adhesives has become an urgent problem to be solved.

In response to the above problems, researchers have proposed many methods for reducing formaldehyde emissions, e.g., the use of formaldehyde scavengers, modifying the adhesives, post-treatment of the finished panels, etc. [3,4]. However, one of the best reported methods is the utilization of biomass materials in adhesive formulations to circumvent the emission of free formaldehyde at the source. Among the many biomass resources, lignin is rich in reserves and contains functional groups such as aliphatic hydroxyl, phenolic hydroxyl, and methoxy groups. It can be modified to synthesize a variety of new polymers, which can effectively replace petroleum-based raw materials for the preparation of adhesives. Today, there are two primary ways of preparing lignin adhesives. One is the blending modification of lignin and trialdehyde resin as a wood-based panel adhesive [5]. This is because lignin has numerous phenolic hydroxyl groups, which leads to its high reactivity, and can be condensed with formaldehyde under certain conditions. Therefore, blending industrial lignin or modified industrial lignin with trialdehyde resin offers the dual benefit of mitigating formaldehyde and phenol content within the resin, thereby diminishing the release of hazardous compounds [6,7,8,9,10,11]. Additionally, this approach enhances the mechanical characteristics of wood-based panels [12,13]. However, while this method effectively reduces the emission of formaldehyde, it falls short of complete elimination of the compound. Another method is to directly use lignin as an adhesive after modification. Related studies have shown that industrial lignin can produce a large number of free radicals after being modified by peroxidase and laccase, and promote its own condensation through free radical coupling to improve its adhesive properties [14,15]. However, peroxidase and laccase enzymes are primarily effective against water-soluble industrial lignin. The wood-based panels prepared by this method exhibit increased susceptibility to water absorption and deformation, thereby compromising their practical utility in real-world applications. Based on this study, the researchers found that DHP with a lignin-like structure can be obtained by treating small-molecule phenols using peroxidase and laccase [16]. The phenoxy radicals on DHP can be coupled with the free phenolic hydroxyl groups on the surface of thermomechanical pulp (TMP) fibers under the catalysis of enzymes [17], thereby effectively improving the wet tensile strength of paper [18,19]. Inspired by these studies, we sought to investigate the potential utilization of lignin-like DHP as a substitute for conventional lignin in order to evaluate its efficacy as a binding agent in the manufacturing process.

In natural wood, lignin and hemicellulose fill in the gaps between cellulose fibrils and form a three-dimensional network structure with fibers to make the fibers intricately connected. In addition, lignin can also form lignin-carbohydrate complexes (LCC) with carbohydrates (mainly hemicellulose) through covalent bonds [20]. The above two traits together give natural wood good physical and mechanical properties [21]. To effectively glue wood-based panel fibers, DHP must satisfy two essential conditions inspired by the structural properties of natural wood. First, it must be able to self-condense to synthesize a polymer during the hot-pressing process, so as to soften at hot temperatures and fill the gaps between the fibers, thus resulting in the physical curing effect of the adhesive [22]. The second is that the DHP and hemicellulose must be able to form an LCC-like covalent bond connection, which connects the fibers more closely. At present, our research group has conducted a simulation experiment on whether DHP can self-condense to synthesize polymers in a hot-pressing environment. The experimental results show that DHP can self-condense into polymers in a simulated hot-pressing environment, indicating the feasibility of DHP glue for use in wood-based panels [23].

Furthermore, this paper will verify whether DHP can react with hemicellulose to form LCC-like polymer compounds under hot-pressing conditions and explore its reaction mechanism. The structure of the reaction product resulting from DHP’s interaction with the glucuronic acid of xylan under hot-pressing conditions was analyzed by infrared spectroscopy and nuclear magnetic resonance. In summary, this paper provides a theoretical basis for the development of green and safe adhesives with high bonding performance.

## 2. Results and Discussion

**Figure 1:**
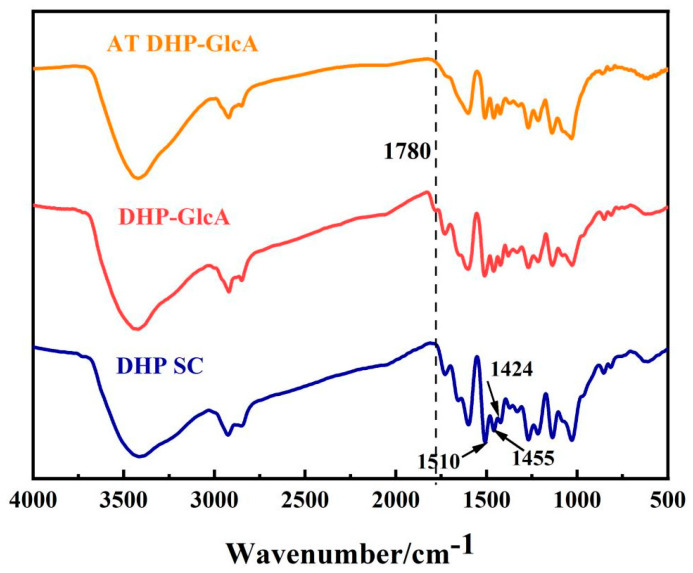
FT-IR spectra of DHP SC, DHP-GlcA, and AT DHP-GlcA (The dotted line is band at 1780 cm^−1^).

The chemical structure of DHP-glucuronic acid complex (DHP-GlcA), alkali-treated DHP-glucuronic acid complex (AT DHP-GlcA), and self-condensed DHP (DHP SC) was detected by various techniques, including FT-IR, CP/MAS ^13^C-NMR, ^13^C NMR, and 2D-HSQC NMR. The infrared spectra of DHP SC, DHP-GlcA, and AT DHP-GlcA are shown in Figure 1. All samples had characteristic absorption peaks of the benzene ring at 1510 cm^−1^, 1455 cm^−1^, and 1424 cm^−1^, which indicated the preservation of the benzene ring scaffold across all three samples [24]. Notably, in comparison to DHP SC, the emergence of a new weak absorption peak at 1780 cm^−1^ in DHP-GlcA suggests the presence of an ester bond, which is in accordance with the findings of Aurore et al. [25]. Furthermore, the disappearance of the 1780 cm^−1^ absorption peak in AT DHP-GlcA confirmed the formation of an ester bond between DHP and glucuronic acid during the thermal condensation reaction.

**Figure 2:**
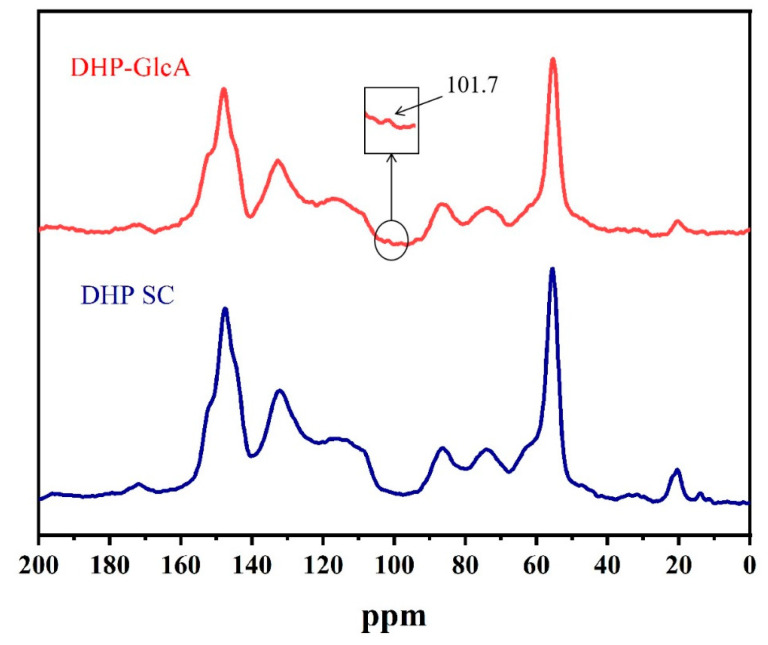
CP/MAS ^13^C-NMR spectra of DHP self-condensation and DHP-glucuronic acid complex.

The CP/MAS ^13^C NMR spectra of DHP SC and DHP-GlcA are shown in Figure 2. Compared to DHP SC, DHP-GlcA showed a new absorption peak at 101.7 ppm. This additional peak likely originated from the C_1_ signal of glucuronic acid [26], indicating the occurrence of a thermal condensation reaction between DHP and glucuronic acid within a 10% volume fraction acetic acid environment at 140 °C. This interpretation is consistent with the above infrared spectrum analysis.

**Figure 3:**
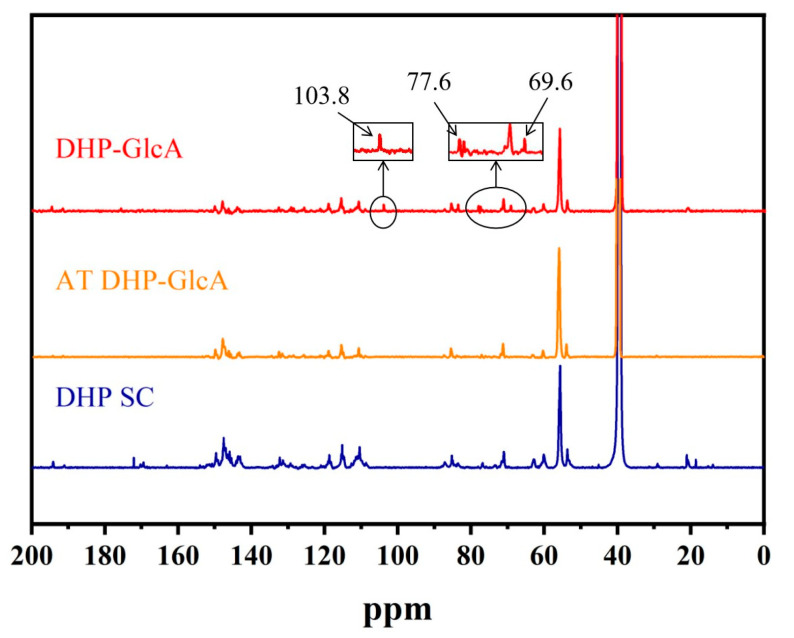
^13^C-NMR spectra of DHP self-condensation, DHP-glucuronic acid–base complex, and DHP-glucuronic acid complex.

Given the less conspicuous nature of the CP/MAS ^13^C-NMR signal, ^13^C-NMR was employed to further characterize the structure of the DHP-GlcA. The ^13^C-NMR spectra of DHP SC and DHP-GlcA are shown in Figure 3. Compared with DHP SC, DHP-GlcA showed new absorption peaks at 103.8 ppm, 77.6 ppm, and 69.6 ppm, corresponding to the C_1_, C_3_, and C_5_ signal peaks within the glucuronic acid, respectively [26]. These signal peaks disappeared after alkali treatment, which indicated the formation of ester bonds between DHP and glucuronic acid. It was concluded that the thermal condensation reaction of DHP and glucuronic acid could generate ester bonds under the above reaction conditions.

**Figure 4:**
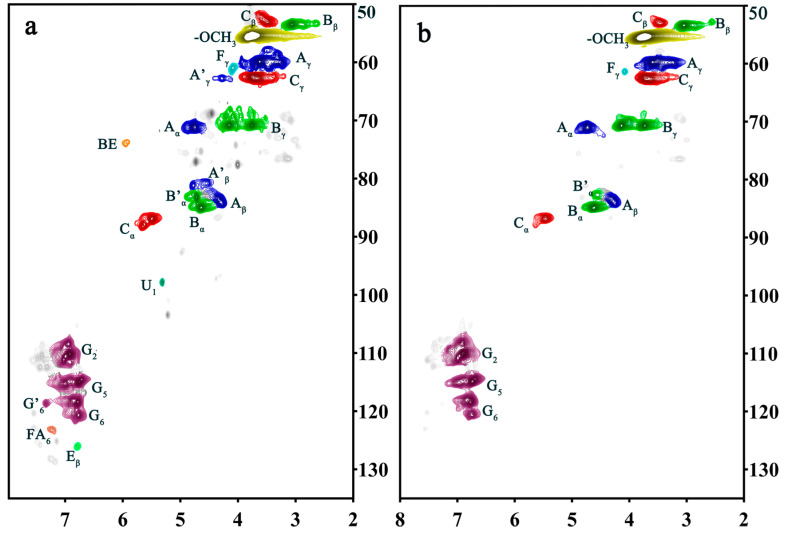
2D-HSQC NMR spectra of the DHP-glucuronic acid complex ((**a**) DHP-GlcA, (**b**) AT DHP-GlcA).

**Figure 5:**
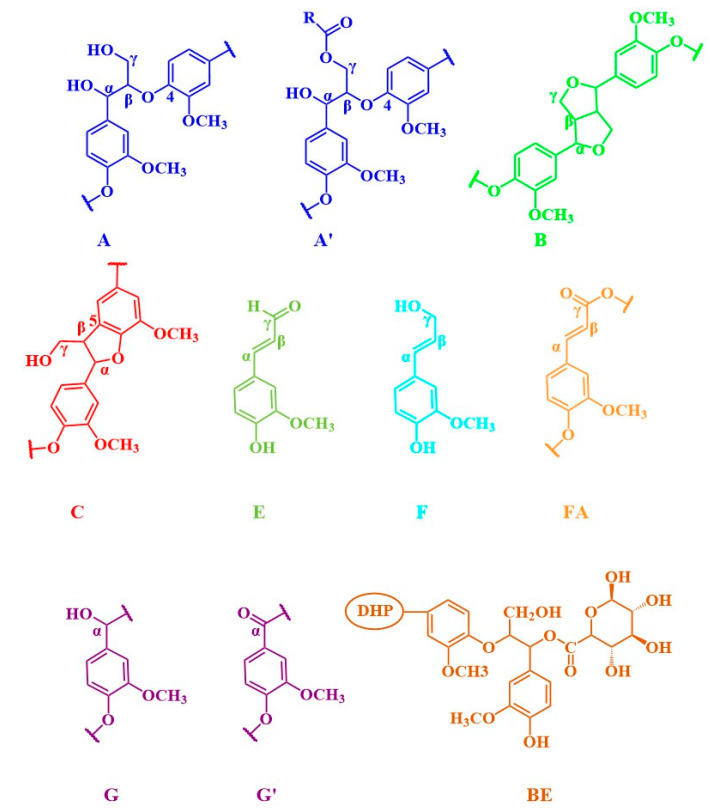
The main connective structures of the DHP–glucuronic acid complex in 2D-HSQC NMR spectra.

**Figure 6:**
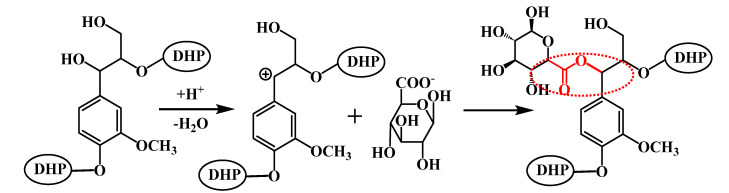
Thermal condensation reaction mechanism of DHP and glucuronic acid.

To substantiate the capability of DHP and glucuronic acid to manifest thermal condensation and form ester bonds under the prescribed reaction conditions, 2D-HSQC NMR analysis was conducted on the ball-milled samples. The 2D-HSQC NMR spectra of DHP- GlcA are shown in Figure 4. The main connective structures of the DHP-glucuronic acid complex are shown in Figure 5. The functional groups of the main signals in the 2D-HSQC NMR spectra were assigned as shown in Table 1 [26,27]. Figure 4a distinctly presents the side chain (β-O-4, β-5, and β-β) and benzene ring (G_2_, G_5_, and G_6_) structures of DHP within DHP-GlcA. This suggests that ball milling enhances the solubility of the complex while preserving its core structure. In Figure 4a, the C_1_-H_1_ signal of glucuronic acid and the ester bond signal at the C_α_ position appeared at 103.40/5.23 ppm (U1) and 73.74/5.94 ppm (BE), respectively, revealing the linkage between DHP and glucuronic acid and the formation of ester bonds during the thermal condensation reaction. After alkali treatment, these two signals disappeared. Based on the ^13^C-NMR analysis, it was inferred that DHP and glucuronic acid are linked via esterification at the C_α_ position during the thermal condensation reaction (Red dotted line in Figure 6). Alkali treatment disrupted the linkage between DHP and glucuronic acid, causing the glucuronic acid to dissociate from the complex. Subsequently, the glucuronic acid was removed during the following washing and centrifugation steps, which resulted in the disappearance of the signal. The related mechanism is shown in Figure 6.

## 3. Methods and Materials

### 3.1. Materials and Reagents

DHP was synthesized based on the previous report [23]. D-glucuronic acid was purchased from Aladdin Industrial Corporation (Shanghai, China). Acetate, toluene, and dimethyl sulfoxide were purchased from Sinopharm Chemical Reagent Co., Ltd. (Shanghai, China). All chemical reagents were analytically pure.

### 3.2. Thermal Condensation of DHP with Glucuronic Acid

The thermal condensation reaction of DHP with glucuronic acid was conducted in accordance with the self-condensation reaction of DHP reported previously [23]. Specifically, 800 mg of glucuronic acid, 400 mg of DHP, and 600 μL of acetic acid solution (10 vt%) were loaded into a closed reactor. The reactor was heated to 140 °C in an oil bath and maintained at that temperature for 25 min. After the reaction, the product was collected by centrifugation, followed by washing with water until the pH value of the filtrate reached neutral and drying under a vacuum.

### 3.3. Ball Milling Treatment of DHP–Glucuronic Acid Complex

The above samples were subjected to milling with ZrO_2_ balls at 600 rpm for 15 h in a planetary ball mill. After the ball milling, the sample powder was flushed with toluene, followed by collection with centrifugation and drying under a vacuum.

### 3.4. Alkali Treatment of the DHP–Glucuronic Acid Complex Powder after Ball Milling

The appropriate amount of the above sample powder and 30 mL of 0.5 mol/L NaOH solution were loaded into a centrifuge tube. After fully stirring and allowing the mixture to stand for 10 h, the product was collected by centrifugation, followed by washing with water until the pH value of the filtrate reached neutral and drying under a vacuum.

### 3.5. Structural Characterization of DHP–Glucuronic Acid Complex

The infrared spectra of the DHP self-condensation product (DHP SC), DHP–glucuronic acid complex (DHP–GlcA), and alkali-treated DHP–glucuronic acid complex (AT DHP–GlcA) were acquired with a Fourier-transform infrared (FTIR) spectrometer (NICOLET 6700, Waltham, MA, USA) ranging from 4000 cm^−1^ to 400 cm^−1^. The Cross Polarization/Magic Angle Spinnin Carbon 13-Nuclear Magnetic Resonance (CP/MAS ^13^C-NMR, Bruker Corp., Karlsruhe, Germany) spectra of the DHP self-condensation and DHP–glucuronic acid complex were determined by superconducting nuclear magnetic resonance spectrometer (AVANCE AV 400 MHz, Bruker, Germany). The Carbon 13-Nuclear Magnetic Resonance (^13^C-NMR) spectra and 2-Dimensional Heteronculear Single Quantum Coherence Nuclear Magnetic Resonance (2D-HSQC NMR) spectra of the DHP–glucuronic acid complex and DHP–glucuronic acid complex after DHP self-condensation and alkali treatment were determined by superconducting nuclear magnetic resonance spectrometer (AVANCE III, Bruker, Germany).

## 4. Conclusions

This work was undertaken to verify whether DHP can react with hemicellulose under hot-pressing conditions to form LCC-like polymer compounds and to explore its reaction mechanism. We prepared DHP–glucuronic acid complexes using DHP and glucuronic acid at 140 °C to simulate the hot-pressing environment of synthetic board molding. Through infrared and CP/MAS ^13^C-NMR, we preliminarily confirmed the occurrence of a thermal condensation reaction between DHP and glucuronic acid under the above reaction conditions, resulting in the formation of ester linkages. Subsequently, a comparison of the ^13^C-NMR spectra of DHP-GlcA before and after alkali treatment further supported this conclusion. In summary, this paper explored the reaction mechanism of DHP with glucuronic acid under simulated hot-pressing conditions, which is helpful for understanding the bonding process between adhesives and components of wood-based panels. This paper lays a theoretical foundation for the application of DHP in formaldehyde-free adhesives.

## Figures and Tables

**Table 1:** Analysis of the 2D-HSQC NMR spectra of the DHP–glucuronic acid complex.

Label	pH = 4	pH = 4 (Alkali Treatment)	Assignments
	δ_C_/δ_H_ (ppm)	δ_C_/δ_H_ (ppm)	
C_β_	52.96/3.49	52.93/3.46	C_β_–H_β_ in phenylcoumaran (C)
B_β_	53.48/3.06	53.38/3.04	C_β_–H_β_ in β-β (resinol) (B)
OCH_3_	55.49/3.77	55.32/3.76	C–H in methoxyls
A_γ_	59.89/3.62 59.99/3.28	59.84/3.25 59.74/3.69	C_γ_–H_γ_ in β–O–4 substructures (A)
F_γ_	60.90/4.10	61.37/4.08	C_γ_–H_γ_ in cinnamyl alcohol end-groups (F)
C_γ_	62.66/3.73	62.56/3.71	C_γ_–H_γ_ in phenylcoumaran (C)
A′_γ_	62.74/4.27	ND	C_γ_-H_γ_ in γ-acylated β-O-4 substructures(A’)
B_γ_	70.78/3.75 70.83/4.51	70.61/3.74 70.68/4.13	C_γ_–H_γ_ in β-β resinol (B)
A_α_	71.19/4.76	71.03/4.73	C_α_–H_α_ in β–O–4 unit (A)
BE	73.74/5.94	ND	α-ester
A_β_	84.00/4.32	84.02/4.29	C_β_–H_β_ in β–O–4 substructures (A)
A′_β_	80.81/4.55	ND	C_β_–H_β_ in β–O–4 linked to G (A)
B_α_	84.96/4.63	84.83/4.62	C_α_–H_α_ in β-β resinol (B)
B′_α_	83.25/4.72	82.68/4.55	C_α_–H_α_ in β-β (B’, tetrahydrofuran)
C_α_	86.89/5.48 87.89/5.64	86.73/5.46	C_α_–H_α_ in phenylcoumaran (C)
U_1_	97.86/5.31	ND	C_1_-H_1_ in 4-O-methyl-α-D-GlcUA
G_2_	108.51/6.94 110.23/6.93	108.31/6.92 110.03/6.90	C_2_–H_2_ in guaiacyl units (G)
G_5_	114.65/6.76 115.25/6.99	114.51/6.74 115.17/7.06	C_5_–H_5_ in guaiacyl units (G)
G_6_	118.40/6.78 120.51/6.77	118.25/6.16 120.55/6.74	C_6_–H_6_ in guaiacyl units (G)
G′_6_	118.52/7.29	118.70/7.27	α C_6_-H_6_ in G-type structural units with oxidized sites
E_β_	125.99/6.79	ND	C_β_–H_β_ in cinnamyl aldehyde end-groups (E)
FA_6_	123.25/7.21	ND	C_6_–H_6_ in ferulate (p-FA)

## Data Availability

Data is contained within the article.

## References

[1] Batiancela M.A., Acda M.N., Cabangon R.J. (2013). Particleboard from waste tea leaves and wood particles. J. Compos. Mater..

[2] Zhang W., Gao Q., Qin Z., Luo J., Li J. (2014). Research and Development of Wood Adhesive in China. China Wood-Based Panels.

[3] Myers G.E. (1989). Advances in methods to reduce formaldehyde emission. Composite Board Products for Furniture and Cabinets-Innovationsin Manufacture and Utilization.

[4] Younesi Kordkheili H., Pizzi A., Niyatzade G. (2016). Reduction of formaldehyde emission from particleboard by phenolated kraft lignin. J. Adhes..

[5] Peng Y., Zhen X., Lu H. (2006). Purification and Application of Lignin in Urea-formaldehyde Resin. Guizhou Chem. Ind..

[6] Alonso M.V., Oliet M., Rodríguez F., Astarloa G., Echeverría J.M. (2004). Use of a methylolated softwood ammonium lignosulfonate as partial substitute of phenol in resol resins manufacture. J. Appl. Polym. Sci..

[7] Alonso M.V., Oliet M., Rodrıguez F., Garcıa J., Gilarranz M.A., Rodrıguez J.J. (2005). Modification of ammonium lignosulfonate by phenolation for use in phenolic resins. Bioresour. Technol..

[8] Khan M.A., Ashraf S.M. (2007). Studies on thermal characterization of lignin. J. Therm. Anal. Calorim..

[9] Khan M.A., Ashraf S.M., Malhotra V.P. (2004). Development and characterization of a wood adhesive using bagasse lignin. Int. J. Adhes. Adhes..

[10] Kharazipour A., Mai C., Hüttermann A. (1998). Polyphenoles for compounded materials. Polym. Degrad. Stab..

[11] Vázquez G., González J., Freire S., Antorrena G. (1997). Effect of chemical modification of lignin on the gluebond performance of lig-nin-phenolic resins. Bioresour. Technol..

[12] Çetin N.S., Özmen N. (2002). Use of organosolv lignin in phenol–formaldehyde resins for particleboard production: I. Organosolv lignin modified resins. Int. J. Adhes. Adhes..

[13] Vázquez G., Antorrena G., González J., Mayor J. (1995). Lignin-phenol-formaldehyde adhesives for exterior grade plywoods. Bioresour. Technol..

[14] Haars A., Kharazipour A., Zanker H., Huttermann A. (1989). Room-Temperature Curing Adhesives Based on Lignin and Phenoloxidases. Adhesives from Renewable Resources.

[15] Hüttermann A., Milstein O., Nicklas B., Trojanowski J., Haars A., Kharazipour A. (1989). Enzymatic Modification of Lignin for Technical Use. Lignin.

[16] Yamaguchi H., Maeda Y., Sakata I. (1991). Application of the dehydrogenative polymerization of vanillic acid to bonding of woody fibers. Mokuzai Gakkaishi.

[17] Lund M., Felby C. (2001). Wet strength improvement of unbleached kraft pulp through laccase catalyzed oxidation. Enzym. Microb. Technol..

[18] Yamaguchi H., Maeda Y., Sakata I. (1992). Applications of phenol dehydrogenative polymerization by laccase to bonding among woody-fibers. Mokuzai Gakkaishi.

[19] Yamaguchi H., Maeda Y., Sakata I. (1994). Bonding among woody fibers by use of enzymatic phenol dehydrogenative polymerization. Mokuzai Gakkaishi.

[20] Pei J. (2014). Lignocellulosic Chemistry.

[21] Bolker H.I. (1963). A Lignin Carbohydrate Bond as revealed by Infra-red Spectroscopy. Nature.

[22] Yang G., Gong Z., Luo X., Chen L., Shuai L. (2023). Bonding wood with uncondensed lignins as adhesives. Nature.

[23] Wang X. (2022). Study on Thermal Condensation Reaction Mechanism of Dehydrogenation Polymer Catalyzed with Acid. Master’s Thesis.

[24] Jiang B., Shen F., Jiang Y., Huang M., Zhao L., Lei Y., Hu J., Tian D., Shen F. (2024). Extraction of super high-yield lignin-carbohydrate complexes from rice straw without compromising cellulose hydrolysis. Carbohydr. Polym..

[25] Richel A., Nicks F., Laurent P., Wathelet B., Wathelet J.-P., Paquot M. (2012). Efficient microwave-promoted synthesis of glucuronic and galacturonic acid derivatives using sulfuric acid impregnated on silica. Green Chem. Lett. Rev..

[26] Wen J.-L., Sun S.-L., Xue B.-L., Sun R.-C. (2013). Recent Advances in Characterization of Lignin Polymer by Solution-State Nuclear Magnetic Resonance (NMR) Methodology. Materials.

[27] Han J., You X., Wang S., Chen C., Yao S., Meng C., Liang C., Zhao J. (2022). Chlorine dioxide oxidation of hemicellulose from alkaline hydrolysate bagasse to remove lignin unit in lignin-carbohydrate complex. Carbohydr. Polym..

